# Design and Characterization of a Powered Wheelchair Autonomous Guidance System

**DOI:** 10.3390/s24051581

**Published:** 2024-02-29

**Authors:** Vincenzo Gallo, Irida Shallari, Marco Carratù, Valter Laino, Consolatina Liguori

**Affiliations:** 1 Department of Industrial Engineering, University of Salerno, 84084 Fisciano, Italy; vgallo@unisa.it (V.G.); vlaino@unisa.it (V.L.); tliguori@unisa.it (C.L.); 2 Department of Computer and Electrical Engineering, Mid Sweden University, 85170 Sundsvall, Sweden; irida.shallari@miun.se

**Keywords:** powered wheelchair, deep neural network, distance measurement methodology, metrological characterization

## Abstract

The current technological revolution driven by advances in machine learning has motivated a wide range of applications aiming to improve our quality of life. Representative of such applications are autonomous and semiautonomous Powered Wheelchairs (PWs), where the focus is on providing a degree of autonomy to the wheelchair user as a matter of guidance and interaction with the environment. Based on these perspectives, the focus of the current research has been on the design of lightweight systems that provide the necessary accuracy in the navigation system while enabling an embedded implementation. This motivated us to develop a real-time measurement methodology that relies on a monocular RGB camera to detect the caregiver’s feet based on a deep learning method, followed by the distance measurement of the caregiver from the PW. An important contribution of this article is the metrological characterization of the proposed methodology in comparison with measurements made with dedicated depth cameras. Our results show that despite shifting from 3D imaging to 2D imaging, we can still obtain comparable metrological performances in distance estimation as compared with Light Detection and Ranging (LiDAR) or even improved compared with stereo cameras. In particular, we obtained comparable instrument classes with LiDAR and stereo cameras, with measurement uncertainties within a magnitude of 10 cm. This is further complemented by the significant reduction in data volume and object detection complexity, thus facilitating its deployment, primarily due to the reduced complexity of initial calibration, positioning, and deployment compared with three-dimensional segmentation algorithms.

## 1. Introduction

The concept of Advanced Driver-Assistance Systems (ADASs) is nowadays applied in an increasing number of fields. Initially, the prevalent field of use has been in the world of self-driving vehicles, particularly in supporting decisions such as obstacle avoidance, lane keeping, and other decisions to avoid accidents [1,2]. ADASs have since been employed in drones for both military and civilian use as a useful aid in avoiding obstacles in flight and supporting the ground operator in operating the aircraft [3].

The development of ADAS has led researchers to focus on several tasks ancillary to the system’s operation, among which are the detection and tracking algorithms on the software side, while for hardware, there has been increased research on imaging sensors, stereo cameras, LiDAR (Light Detection and Ranging), and radar. In addition to the automotive field, the computer vision hardware developed has found use in the fields of agriculture, archaeology, biology, geology, and robotics. Among other things, it has been made possible to scan buildings, objects, terrain, and others, obtaining accurate three-dimensional models in less time than using other techniques [4]. The common aim of the application of these technologies to different fields concerns the automation of application-specific processes: the design of these smart sensor nodes is often with the intent of producing a large amount of data (big data) in order to employ neural networks for the extraction of features of interest [5,6]. These techniques have also found applications in the development of remotely controlled vehicles, which, thanks in part to the development of new artificial intelligence techniques, have succeeded in automating the control of small electric vehicles and robots, used both in domestic settings and in risky situations, such as in bomb disposal [7].

In this article, we focus on the automation of PWs, which has greatly improved the quality of life for people with disabilities by facilitating the wheelchair-driving approach while also providing more independence from caregivers. PWs have already greatly increased the independence of people who cannot move the wheelchair under their own power using external input commands such as specific joysticks; however, they still present limitations in ease of riding, especially for people who have reduced reflexes and less awareness of their surrounding space [8].

Recent work [9] tried to overcome the physical limitation of the PW drivers, focusing on the analysis of their nerve signals, using deep learning, and trying to interpret these stimuli as driving inputs. Other work focused on the vision-based PW piloting method [10], still with the aim of identifying the PW driver’s steering intention, by analyzing their head tilt. Thus, once more, the person with a disability needs to operate the chair independently, indicating the direction of motion by their own will. Alternatively, multiple solutions for the autonomous navigation of these devices in controlled environments have been proposed in the literature [11,12,13,14,15,16], such as combining three LiDAR sensors and an omnidirectional camera placed on a pole [17,18]. The main focus of all these methodologies has been to have the wheelchair follow the caregiver so that it can be used even for people with severe disabilities, such that any nervous or physical stimulus to drive the PW is prevented. In these works, the caregiver distance has been measured using LiDAR sensors, which measured the human chest profile represented by an ellipse, while the omnidirectional camera distinguished the caregiver from the other people nearby. Despite the good detection results, it has been difficult to integrate all of the required sensors and electronic systems into the wheelchair due to limited installation space for an additional embedded system and a limited power supply. Furthermore, mounting the camera on a pole altered the ergonomics and appearance of the PW, rendering it unsuitable for commercial use. An alternative camera placement has been demonstrated in [19], where a stereo camera has been mounted on the PW’s armrest. In this case, the lower camera placement would not allow for measuring the entire human body shape due to limitations in the camera’s field of view. As a result, caregiver detection has been accomplished by detecting the caregiver’s legs.

An alternative recent approach [20], on the contrary, is based on the use of a single camera, pointed in the direction of travel of the PW, with the goal of classifying obstacles in its path. However, this methodology does not make distance measurements and, therefore, does not allow the application of an accurate path control logic. Nevertheless, the idea of using a simple monocular camera to control the PW could be an important element in simplifying the setup of the PW itself due to the greater simplicity of the distance estimation algorithms compared with those based on 3D clusters but also in strong cost savings, to date amounting to about an order of magnitude. Despite this, the use of a single monocular camera does not come without problems. In fact, other setups presented in the literature [21], which can be used to cut down on the cost of hardware needed for automatic control of a PW, consisted of a stereo camera and an RGB camera to detect and track the feet of the caregiver based on the Tiny YOLO (You Only Look Once) neural network. In this case, although the use of Convolutional Neural Networks brings great robustness and accuracy of results, in accordance with what was demonstrated in [22], the setup employed had this critical issue: it required an additional depth camera, whether based on LiDAR or stereo camera technology, because distance estimation with the monocular camera exhibited very limited accuracy.

The methodologies presented strongly highlighted how the measurement accuracy of the caregiver distance from the PW is a crucial element that can potentially compromise the safety of the vehicle and surrounding pedestrians, preventing the implementation of a safe and robust path control logic. However, to date, this type of analysis for systems based on computer vision and deep learning is almost completely absent in the literature. The main research interest is instead focused on improving automatic feature extraction and, thus, in the architecture of these systems; for this reason, the parameters used to measure the performance of deep learning algorithms are never valid from a metrological point of view.

Considering the points discussed, this work proposes the following contributions:

- Development of a measurement methodology for autonomous driving of a PW based on a monocular camera and an object detection neural network;
- Creation of an object detection dataset that can independently classify whether the foot present in the scene is resting on the ground or not, with the aim of mitigating parallax errors due to the use of a monocular camera
- Metrological characterization of the measurement system. This is achieved by calibrating the camera used for acquisitions, correcting for systematic effects on the instrument’s calibration curve, assessing its uncertainty, and evaluating the entire instrument’s uncertainty;
- Evaluation of the metrological performance for the distance to caregiver measured with the proposed method compared with LiDAR and stereo-camera-based systems;
- Deployment of the proposed system on a PW in a real-case scenario.

Section 2 introduces the state of the art of object detection and the issues that arise from the metrological point of view. Section 3 and Section 4 present the camera setup, the dataset construction, and the network training. Section 5, Section 6 and Section 7.1 outline the calibration of the instrument and its metrological characterization. Finally, Section 7.2 focuses on the experimental deployment of the proposed system in a real-case scenario.

## 2. Object Detection State-of-the-Art

The first step in measuring the caregiver’s distance from the wheelchair is to identify a body part of the caregiver. In this article, we focus on foot detection because of the camera positioning and the ease of detection compared with legs or torso due to more distinct features. Object detection algorithms that can be used in the scenario under consideration can be based on two-dimensional images or three-dimensional images. This difference comes from the type of imaging technology used to acquire the object of interest, which can be a monocular RGB camera or a depth camera based on LiDAR or stereo vision technology.

### 2.1. Depth Images

In designing a vision-based system for measuring distance, depth imaging sensors such as LiDAR or stereo cameras would be the intuitive choice. They produce depth images relying on point cloud data, which are clusters of pixels placed in a three-dimensional space based on the distance of the object at the time of acquisition. Object detection in this type of image is difficult because it first requires a denoising operation, followed by the complexity of extracting features, considering the lack of explicit, neighboring information. Several traditional image processing techniques can be applied, such as Support Vector Machine (SVM), K-Nearest Neighbors (KNN), Multilayer Perceptron, Logistic Regression, and a revised version of the Histogram of Oriented Gradient technique, called 3DHOG [23,24]. Deep-learning-based techniques have also been presented in the literature, where some approaches may include an automatic search for patterns of interest in the point cloud or the use of R-CNNs for proposing three-dimensional regions of interest [25,26]. However, one major drawback of these technologies is the significantly large volume of data followed by high computational demand, as well as high sensor cost, which makes them a suboptimal choice for resource-constrained smart sensor nodes.

### 2.2. Two-Dimensional Images

Regarding two-dimensional images, the state of the art for object detection based on deep learning is Convolutional Neural Network (CNN)-based networks. CNNs are artificial feed-forward neural networks inspired by the animal visual cortex, where the neurons operate as local filters in space, helping to detect meaningful spatial correlations in images. The brain then uses these relationships to identify objects and environments [27,28,29]. These algorithms that rely on large data volumes are becoming viable due to the increasing computational and storage power.

In recent years, these techniques have outperformed traditional computer vision techniques, such as Histogram of Oriented Gradients (HOG) [30] algorithms, image segmentation, SVM, and other filtering operations [31], due to the increased computational power of the devices used for training. Traditional techniques were characterized by high specificity, resulting in a complex design process, especially when there are several objects of interest. Instead, in the design of CNNs for a given application, constructing a variegated dataset is a prerogative of high robustness in detecting objects of interest.

The types of neural networks used for object detection fall into two main categories:

- Object segmentation, where each pixel in the image is classified according to whether it belongs to the foreground or background;
- Positional object detection, where the object is identified either by multiple classification tasks performed with sliding windows or by algorithms based on probability areas (YOLO).

Segmentation carried out with Regional Convolutional Neural Network (R-CNN) types of networks [32] is computationally demanding because, first, the image is segmented in several regions that share similar characteristics, and afterwards, each segmented region of the image is fed to the CNN for classification [33]. Considering that these segmented regions would overlap between them, then every image is processed more than once from the CNN. The most widely used techniques to date are always based on deep learning and include R-FCNs (Region-based Fully Convolutional Networks) [34], RetinaNet [35], SSD (Single-Shot MultiBox Detector) [36], and DSSD (Deconvolutional Single-Shot Detector) [37,38].

A YOLO network is able to reduce the object detection problem to a single regression problem, directly from the image pixels to the coordinates of the bounding boxes related to the identified objects. This makes it possible to have a high number of frames per second in inference. In [39], it is reported that YOLO, in its old version (v3), provides a lower detection accuracy value than SSD and RetinaNet by about two percent, with a reported inference time down to one-third of the other two networks. The network used in this article is YOLOv5, which has significantly better performance than YOLOv3 [40]. These considerations promote YOLOv5 as the best object detection network in the literature, considering both inference time and detection accuracy.

## 3. Camera Setup and Dataset Definition

The PW navigation system is focused on the detection of the caregiver’s feet, which allows them to reduce the camera’s field of view compared with detecting the caregiver’s whole body. Subsequently, this choice has advantages for the use of YOLO as an Object Detection algorithm: especially, it simplifies the dataset creation and reduces the background noise, averting false-positive detection.

Foot detection allows the authors to develop an approximate distance measurement system without calibrated references in the camera’s field of view. In fact, knowing the height from the ground of the camera and the framing angle with respect to the ground, Equations (1)–(3) have been applied,

(1) $$\phi =\tan^{-1}\left(\frac{C-C_{c}}{f}\right)$$

(2) $$\beta =\tan^{-1}\left(\frac{R-R_{c}}{f}\right)$$

**Figure 1:**
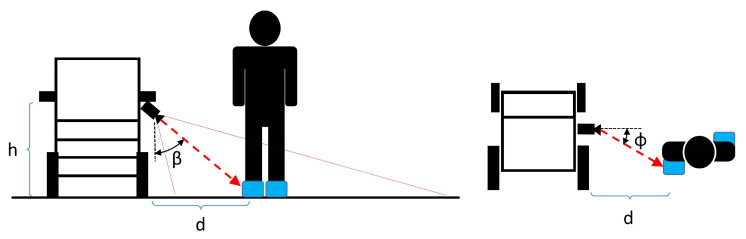
Caregiver distance measurement setup [21].

**Figure 2:**
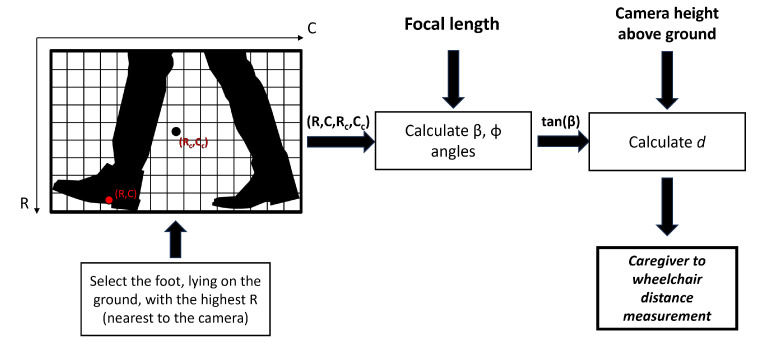
Flowchart of the proposed methodology with the illustration of row and column extraction from the image.

(3) d=htanβ

where *R* is the row coordinate of the center of gravity of the detected foot, $R_{c}$ is the center row of the camera, *C* is the column coordinate of the center of gravity of the detected foot, and $C_{c}$ is the center column of the camera, all expressed in pixels. In addition, *f* is the focal length of the optics employed, β is the camera mounting angle with respect to the ground, and *h* is the height of the camera with respect to the ground. A schematic representation of the measurement setup is shown in Figure 1. Instead, a graphical representation of the rows and columns of the acquired image is shown in Figure 2. This image also shows the flowchart of the proposed methodology The dataset used for YOLO training is based on more than 4000 images taken with the measurement setup described above. The dataset consists of images taken in multiple environments, both indoors and outdoors, under different illuminations. These images contain RGB visual data frames on 3 channels and depth frames in the form of 1-channel depth maps, alongside images that are a combination of RGB and depth in the form of a 4-channel stream.

**Figure 3:**
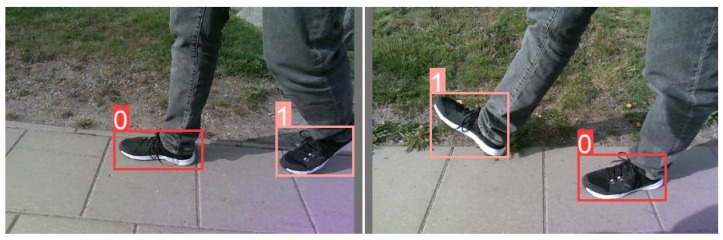
Images from the improved dataset. Pink bounding boxes identify feet in the air and red bounding boxes feet on the ground.

The main limitation of this approach relates to the way in which distance is calculated from the relative position of the foot in the scene; in fact, the algorithm works correctly only if the foot under consideration is resting on the ground, otherwise, the trigonometric formulas defined earlier, in particular Equation (3), are no longer valid. For these reasons, we modified the existing dataset to create two classes, 0 and 1, respectively, for the foot on the ground and the foot up scenarios. Therefore, we overcome the problem by relying on the classification of the neural network and only using the bounding box of the foot on the ground in the distance calculation. An example of the new dataset is given in Figure 3. Upon completion of the new classes, data augmentation was applied for the training only, tripling the number of images to about 12,000 images. The data augmentation applied was a random rotation of the images at an angle between −5° and +5°.

## 4. Training Results

The YOLO neural network was trained on 70 epochs, with a lower limit of 0.2 for the IoU (Intersection over Union) in Equation (8). Furthermore, 70% of the dataset was used for training and 30% for validation. The parameters used to analyze the performance of the YOLO neural network are Precision (4), Recall (5), mAP (6), and Confidence Score (7),

(4) $$Precision=\frac{TP}{TP+FP}$$

(5) $$Recall=\frac{TP}{TP+FN}$$

(6) $$mAP=\frac{1}{n}\sum_{k}^{n}AP_{k}$$

(7) $$Confidence\;Score=\mathrm{Pr}\left({\mathrm{Class}}_{i}\right)*{\mathrm{IOU}}_{\mathrm{pred}}^{\mathrm{truth}}$$

(8) $$IoU=\frac{Area\;of\;Overlap}{Area\;of\;Union}$$

where TP are the true positives, FP are the false positives, and FN false negative. All these components are calculated when the IoU between the inference and ground truth bounding boxes is greater than 0.5. $AP_{k}$ stands for Average Precision, calculated for each image *k*. This value can be computed using 0.5 as the IoU threshold or an average between 0.5 and 0.95, which is useful to visualize the performance for more accurate localization of the object in the image.

The trained model was then tested on 300 images not used in the training phase. The model was then evaluated by analyzing the Precision–Recall curve, which reports the Precision and Recall values as confidence varies in the detection of the YOLO network. In addition, the $F_{1}$ curve reports the variations in $F_{1}$ as the confidence varies, which is presented in Equation (9).

(9) $$F_{1}=2\cdot \frac{Precision\cdot Recall}{Precision+Recall}$$

**Figure 4:**
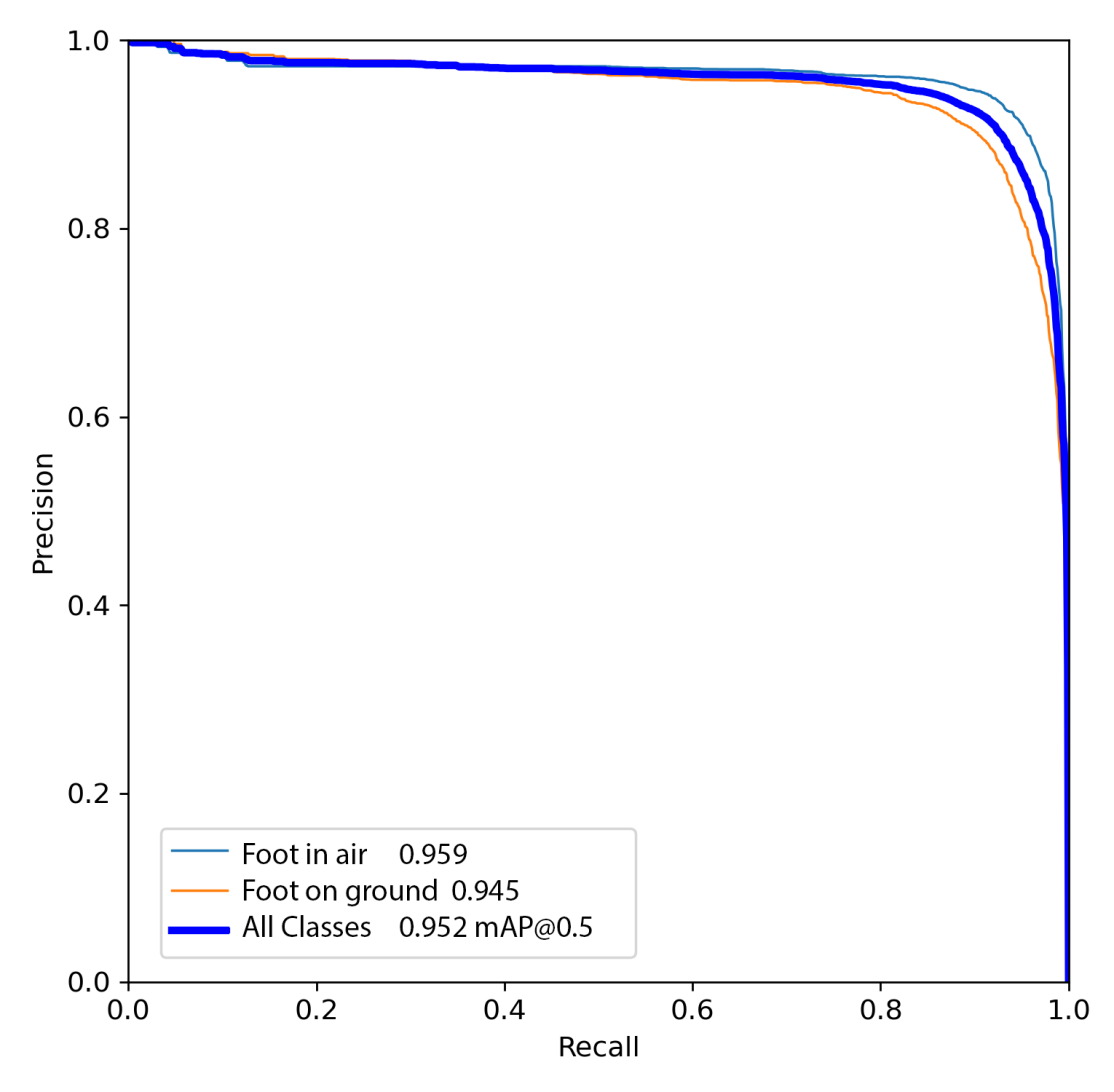
Precision–Recall curve for the testing set: blue line represents the foot-on-ground class and the orange line foot-in-air.

**Figure 5:**
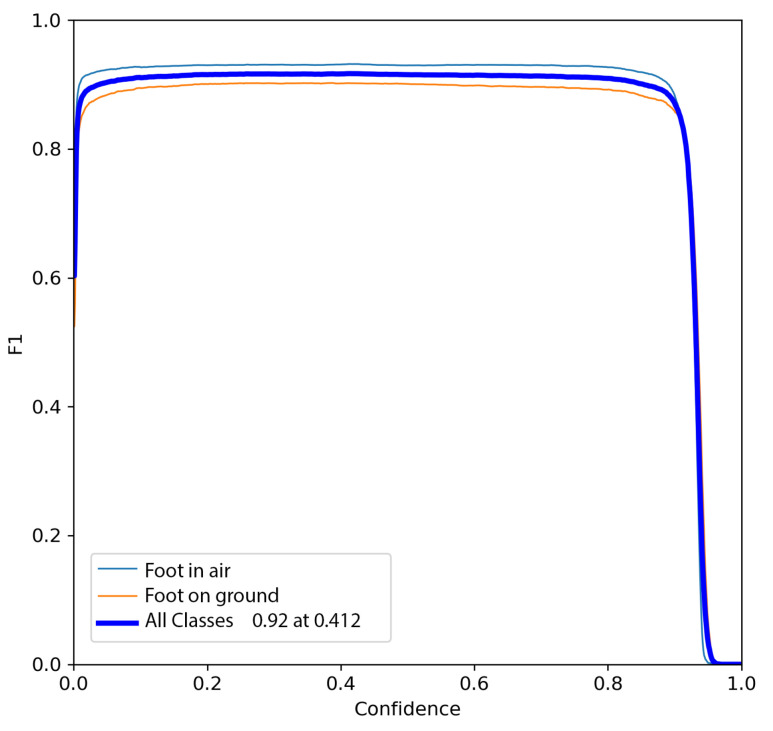
F1 curve for the testing set: blue line represents foot-on-ground class and orange line foot-in-air.

The results of the testing are shown in Figure 4 and Figure 5. Testing resulted in a maximum F1-score of 0.9, achieved with a confidence score between 0.1 and 0.9, a satisfactory result along with Precision and Recall, both exceeding 0.9. However, from the inference results, and thus from knowing only the coordinates of the center of mass of the bounding boxes, it is not possible to measure the distance of the caregiver from the wheelchair since this also depends on the intrinsic parameters of the camera, as demonstrated in Equations (1) and (2). For these reasons, the camera was calibrated.

## 5. Instrument Calibration

### 5.1. Camera Calibration

The most used method for extracting intrinsic camera parameters today, which also allows the correction of distortions in the image, is Zhang’s calibration [41]. The calibration procedure in question requires multiple shots of a checkerboard-shaped target with squares of known size. Thus, after fixing the camera at the position defined by the measurement setup, it was necessary to acquire more than eight images with the target placed at different distances, always consistent with the distances and positions of the objects to be identified.

The calibration was performed with MATLAB 2023a software, where in addition to performing an image distortion correction, we also calculated the intrinsic and extrinsic parameters of the camera under test and the reprojection error. This error is the distance, in pixels, between the detected and the corresponding reprojected points, which are the corners of the calibrating checkerboard [41]. At the end of the calibration, which reported a reprojection error of fewer than 0.5 pixels, it was then possible to obtain data on the intrinsic parameters of the camera employed. The results of the camera calibration procedure are reported in Table 1. Thanks to this calibration, several distortion effects in the peripheral parts of the image were also corrected.

### 5.2. Calibration Curve

Initial metrological validation of the designed system consisted of a multistep calibration over the entire measurement range. In order to carry this out, it was necessary to arrange a special setup consisting of the wheelchair and several colored strips on the ground placed at different distances from the wheelchair. Distance measurement was performed with a measuring tape, with an uncertainty of less than 1 mm.

**Figure 6:**
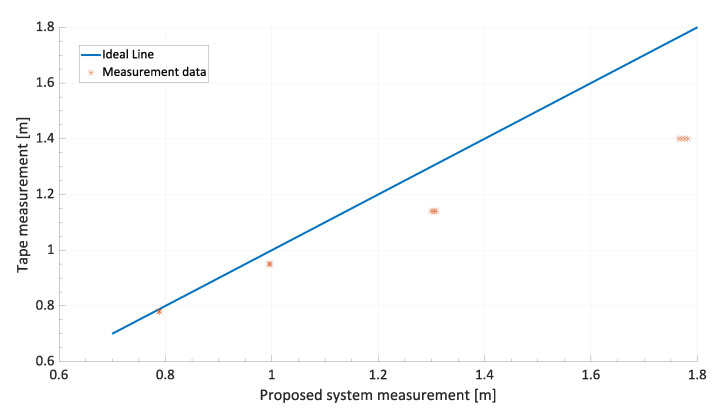
Plot of the uncorrected calibration curve where the strong nonlinearity and gain error compared with the ideal line is noticeable.

Multiple shots of the feet placed statically at different distances were taken at predefined time intervals, repeating the measurement 50 times. In this way, in addition to assessing the residual error of the instrument, it was possible to evaluate the uncertainty of the measurement. This analysis is carried out in Section 6. As for calibration, the distance references were placed at 0.780 m, 0.950 m, 1.140 m, and 1.400 m. The calibration curve, shown in Figure 6, was then plotted.

The results of the calibration curve show that the deviation of the measured values from the ideal calibration line is much greater for values close to the full scale of the instrument. The systematic error contribution to the full scale of the instrument is mainly due to Equation (3). In particular, when the caregiver moves away from the wheelchair, the angle β, reported in the equation, increases to over 60°. For these angles, however, the slope of the tangent function is very high, which has the effect that a small systematic error made by the YOLO network in identifying the correct row *R* of the foot’s center of mass generates a larger distance measurement error.

**Figure 7:**
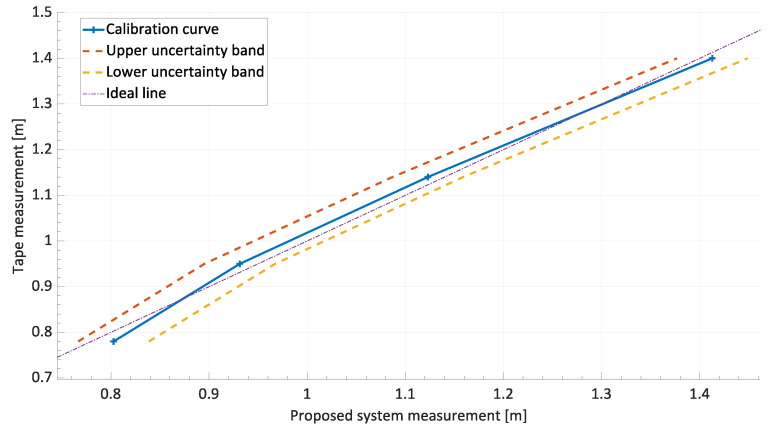
Corrected calibration curve ($R^{2}=0.994$).

Systematic errors were then corrected by adjusting the gain errors and offset errors of the calibration curve in order to achieve a bisector curve of the first quadrant. The new calibration curve, obtained after correction based on linear regression, is shown in Figure 7. The calibration curve uncertainty bands calculated with a confidence level of 95% were also plotted in the figure. At the end of the procedure for the correction of systematic effects, the maximum error, defined as deviation from the ideal calibration line, was calculated as 2.2 cm.

## 6. Metrological Characterization

The step following the correction of the systematic errors of the proposed distance measurement system is the metrological characterization. The aim was to analyze the uncertainty contributions arising from the measurement setup. Measurement uncertainty and the maximum displacement between the measured value and the reference value estimation were also carried out. As it is possible to observe in Figure 6, the full-scale distance measurement is affected by higher uncertainty, as proved by the greater scattering of measurement samples on the right-hand side of the figure.

To investigate the cause of this uncertainty, we analyzed the issue firstly from a theoretical point of view, using a Type B uncertainty propagation. For this purpose, the ISO GUM standard, which regulates the analysis of measurement uncertainty and the study of its contributions, was used [42]. In particular, the guide defines the General Law of Uncertainty Propagation that relates different contributions of uncertainty in relation to their weight within an analytical model. The uncertainty propagation of Equation (10) is formulated in Equation (11) and involves the calculation of sensitivity coefficients, which are the partial derivatives of the analytical model variables.

(10) z=f(x,y)

(11) $$u_{z}=\sqrt{{\left(\frac{\partial f}{\partial x}\right)}^{2}\cdot u_{x}^{2}+{\left(\frac{\partial f}{\partial y}\right)}^{2}\cdot u_{y}^{2}+2\rho \frac{\partial f}{\partial x}\frac{\partial f}{\partial y}u_{x}u_{y}}$$

Through the ρ coefficient of correlation, the general law additionally accounts for correlations between the variables under study.

On this theoretical basis, it was then decided to propagate the error on Equation (3). The resulting formulation of uncertainty propagation is reported in Equation (12), as the measures of the angle β and camera height from the ground *h* were not correlated.

(12) $$u_{d}=\sqrt{\frac{h^{2}}{cos^{4}\left(\beta \right)}\cdot {\left(u_{\beta }\right)}^{2}+\tan^{2}\beta \cdot {\left(u_{h}\right)}^{2}}$$

The uncertainty of the height above the ground of the camera *h* was lower than the uncertainty of the beta angle, as it was measured with a dedicated measurement tape. Hence, Equation (12) can be rewritten as Equation (13).

(13) $$u_{d}\approx \frac{h}{cos^{2}\left(\beta \right)}\cdot u_{\beta }$$

This analysis is also known as the Sensitivity Analysis, since, according to the ISO GUM standard, the equation defines the response of the modeled system to small perturbations (δ). Thanks to this theoretical analysis, it can be concluded that for values of β greater than 45°, the $u_{d}$, that is, the uncertainty of the caregiver’s distance measurement from the wheelchair, increases significantly. This analysis showed results compatible with the ones shown in the curve of Figure 6 because, for distances greater than 1.00 m, the angle β has been determined to be greater than 60°. Thus, for large distances, the predominant uncertainty contribution is not caused by the neural network but by the parallax error formalized in the trigonometric equations. Therefore, it was decided to proceed with the experimental estimation of this uncertainty using a Type A approach, as defined by the ISO GUM standard.

### 6.1. Measurement Uncertainty Estimation

Experimental uncertainty analysis of the distance measurement was carried out by repeated measurements, 50 times for each of the calibration points, which were set at these distances: 0.780 m, 0.950 m, 1.140 m, and 1.400 m. In fact, uncertainty, as defined by ISO GUM [42], is indeed important to verify the stability of the instrument for measurements made over a short period of time without changing the measuring instruments used.

Based on the collected measurements, a uniform type distribution was assumed. Calculated Δ values are given in Table 2, where 2·Δ is defined in Equation (14) for each calibration point *i* and measurements $\hat{x}$.

(14) $$2\cdot \Delta_{i}=\max\left(\hat{x_{i}}\right)-\min\left(\hat{x_{i}}\right)$$

To evaluate the uncertainty, the standard deviation of the distribution of observations was calculated according to Equation (15), as defined in [42].

(15) $$u_{i}=\frac{\Delta_{i}}{\sqrt{3}}$$

Therefore, the maximum uncertainty was observed for a distance of 1.4 m. The uncertainty contribution of the correction applied was equal to the uncertainty of the reference measurement, which is less than 0.001 m. The overall uncertainty expanded to a confidence level of 95%, which also takes into account the uncertainty of the correction, was $U_{1.4m}$ = 0.010 m.

### 6.2. RMSE and Maximum Error

Knowledge of the different true values of the distance between the caregiver and the PW, which were used in the calibration phase of the instrument, made it possible to calculate two synthetic parameters for evaluating the accuracy of the proposed measurement instrument. A first analysis was performed by calculating the Root-Mean-Square Error (RMSE) overall value, thus taking into account the measurements made throughout the operational range of the instrument, as reported in Equation (16),

(16) $$RMSE=\sqrt{\frac{1}{N}\sum_{i=0}^{N}{\left(x_{i}-\hat{x_{i}}\right)}^{2}}$$

where $x_{i}$ is the true value of distance, while $\hat{x_{i}}$ is the value measured by the proposed instrument. *N* is the numerosity of the whole sample of measurements, and the resulting RMSE is equal to 0.003 m.

The calculation of RMSE is not sufficient to fully define the accuracy of a measuring instrument, as this value does not allow the performance to be analyzed in relation to the measurand. To overcome this problem, it was decided to estimate the instrument class; a parameter estimates the maximum displacement between the measured value and the reference value, also taking into account the full scale of the instrument, as defined in Equation (17).

(17) $$Class\;of\;Accuracy=\frac{|\hat{x_{i}}-x_{i}|}{FS}\cdot 100$$

Again, $\hat{x_{i}}$ stands for the single observation of the proposed instrument and $x_{i}$ for the single reference observation. In addition, in the equation, there is the term FS, which stands for Full Scale, which is the maximum distance value that can be measured by the instrument. This parameter then allows the absolute error of the measurements and the measuring range of the instrument to be related. The result of (17) is rounded to the nearest 0.5 to define the class of the instrument. The class of the proposed instrument was calculated for the worst case of absolute error for all measurements made. Therefore, the proposed instrument can be defined as class2.

## 7. Discussion

### 7.1. Metrological Performance Comparison

The results presented report good metrological performance after correction for systematic effects. In particular, the proposed system demonstrated good temporal stability and an instrument class2. To validate the results and verify the goodness of this monocular camera-based distance measurement methodology, we compare the proposed method with a LiDAR-based measurement system and one based on a stereo camera. To enable the comparability of these technologies, the same measurement setup has been used for all the different camera types. As for the LiDAR camera, it has been possible to perform the acquisitions directly and in the exact same scenario proposed in Section 3, replicating all the steps of calibration, correction of measurements, and evaluation of instrument class and uncertainty. More in detail, the Depth Camera used for comparison was an Intel RealSense D455 based on stereo vision. The deployed LiDAR, on the other hand, was an Intel LiDAR Camera L515. As for the comparison with other depth measurement cameras, it was decided to take the metrological parameters of the Depth Camera from the [43] camera datasheet.

In particular, it can be seen from the table that the proposed system performed worse than LiDAR and better than the stereo camera. This is shown by both uncertainty and maximum error: the proposed system can be categorized into a class2 instrument, the LiDAR in class1.5, and the stereo camera in class2. Thus, the performance of the stereo camera and LiDAR camera is comparable to that of the proposed system. However, the use of these two instruments within a measurement setup such as the one under consideration can present serious difficulties. First, the cost of these cameras must be taken into consideration, which is at least ten times the cost of the monocular camera used in the proposed measurement system. Furthermore, LiDARs are very sensitive to solar radiation when deployed outdoors, resulting in a possibility of compromising some of the measurements made. Second, the complexity of the object detection algorithms applied to these types of images must be considered; in fact, as described in the introduction and state-of-the-art sections, the object detection algorithms for these cameras must work in a three-dimensional spatial domain, applying complex techniques to identify points in space belonging to the same cluster, such as the 3D Histogram of Oriented Gradient. The rapid development of object detection neural networks such as YOLO and the continued optimization of computational weight have actually made it more advantageous to use detection techniques on a two-dimensional domain than a three-dimensional one. This is of significant importance in the proposed system, as it has to be embedded in the PW and has to measure the distance to the caregiver in real time in order to maintain safe autonomous navigation. The results of the comparative assessment are summarized in Table 3.

### 7.2. Use Case Scenario Deployment

**Figure 8:**
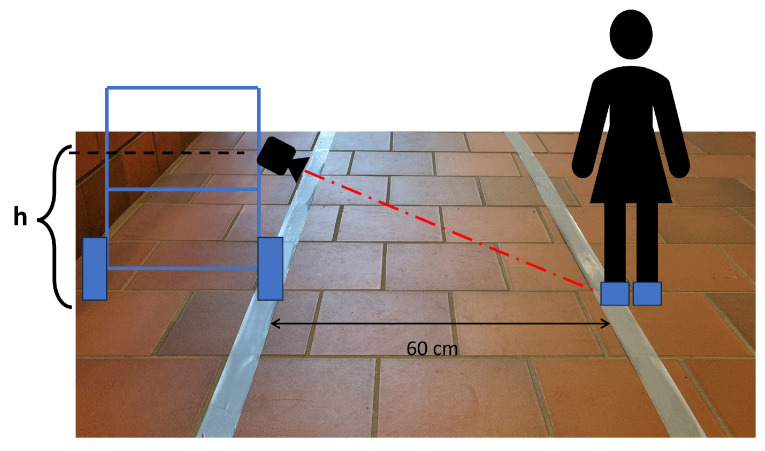
Diagram of the use case scenario test. The figure shows the path of the wheelchair and caregiver employed in the experiment.

**Figure 9:**
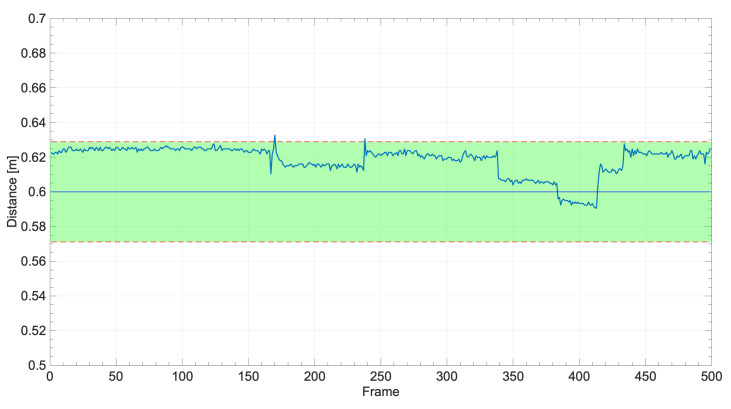
Experimentally measured distances. The green area is the reference measurement with a 68% confidence level.

**Figure 10:**
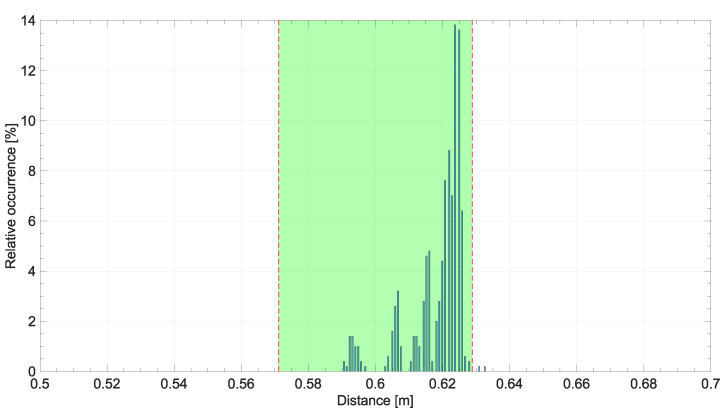
Histogram of the experimentally measured distances. The green area is the reference measurement with a 68% confidence level.

In order to evaluate the potential of the proposed methodology, an experimental setup was used to verify the metrological performance in a real-world application setting. In contrast to previous tests, the conditions of this one were designed to dynamically verify the caregiver’s distance from the PW in a consistent way with what would occur with a physical prototype. For this experimental deployment, an indoor, dimly lit pathway was set up in which the PW and caregiver were placed at a predetermined distance. This distance was measured with a reference meter at different points on the track, the length of which was less than 30 m. To indicate the correct positions for the PW and caregiver to hold during the test, two tapes were placed on the ground at the measured reference distance, as visible in Figure 8. A manually controlled PW was used for deployment. The vision system employed consisted of a U-Eye camera UI-1220LE-M-GL and a Raspberry Pi 5 deputed to image processing, YOLO neural network inference, and subsequent measurement of the caregiver’s distance from the wheelchair. The reference distance was set at 60.0 cm with a standard uncertainty of 2.9 cm. This was mainly due to the thickness of the tape used on the floor. Real-time images were acquired for the test, and the frames were discarded with the foot closest to the chair not fully resting on the ground. According to the classification result of the trained neural network model, the conversion to meters was then performed, and the correction described in Section 5 was applied. The results of the experimental deployment are shown in Figure 9. In the plot, it can be noted that the distance measurement always falls within the standard uncertainty range defined by the reference measurement. The robustness of the methodology is also reflected in the distribution plot of the measurements shown in Figure 10, in which it is possible to assess that almost all measurements fall within the defined confidence interval of the reference measure.

The performance of the embedded system on which they were within acceptable times for real-time execution is as follows: specifically, the average measurement time per single frame was between 385 ms and 395 ms. In conclusion, the experimental deployment of the proposed methodology demonstrated excellent metrological performance in measuring caregiver distance from PW in a real-life deployment scenario. The methodology processing time and the simplicity of the setup arrangement were in line with expectations and suitable for a real-world prototype deployment.

## 8. Conclusions

In this work, the effectiveness of a new distance measurement methodology based on a monocular RGB camera has been demonstrated. The methodology finds applicability in the context of autonomous navigation of Powered Wheelchairs, enabling people with severe motor disabilities to use this type of wheelchair. In conclusion, the following can be stated:

- The methodology finds applicability in the context of autonomous navigation of Powered Wheelchairs, enabling people with severe motor disabilities to use this type of wheelchair.
- Compared with object detection techniques for three-dimensional point clusters, overcoming their limitations and difficulties, the proposed measurement methodology proved less complex in hardware set-up and software deployment.
- The metrological performances obtained by the proposed system have been comparable with those of methodologies based on LiDAR and stereo cameras, making the proposal suitable for implementation in the autonomous navigation setup of future Powered Wheelchairs, optimizing design and costs, and facilitating their diffusion into the market.

Future developments will involve the design of a PW control system based on caregiver-related distance measurements. 

## Figures and Tables

**Table 1:** Intrinsic camera parameters with standard uncertainty.

	Column Value	Row Value
Focal Lenght (Pixels)	860.3±13.1	792.2±6.6
Principal Point (Pixels)	369.5±5.9	239.4±7.9
Radial Distorsion	0.1±0.0	−0.3±0.0
Image Size	1280	720

**Table 2:** Uniform distribution analysis for each calibration point.

Calibration Points	2·Δ
0.780 m	0.001 m
0.950 m	0.003 m
1.140 m	0.009 m
1.400 m	0.016 m

**Table 3:** Metrological performances comparison.

	Proposed System	LiDAR	Stereo Camera
Maximum absolute error (m)	0.022	0.019	0.028
Expanded uncertainty (CL 95%) (m)	0.010	0.005	0.016
RMSE (m)	0.003	0.001	0.005
Instrument Class	2	1.5	2

## Data Availability

No new data were created or analyzed in this study. Data sharing is not applicable to this article.
